# Jellyfish on the menu: mtDNA assay reveals scyphozoan predation in the Irish Sea

**DOI:** 10.1098/rsos.171421

**Published:** 2017-11-29

**Authors:** Philip D. Lamb, Ewan Hunter, John K. Pinnegar, Simon Creer, Richard G. Davies, Martin I. Taylor

**Affiliations:** 1 School of Biological Sciences, University of East Anglia, Norwich Research Park, Norwich, Norfolk NR4 7TJ, UK; 2 School of Environmental Sciences, University of East Anglia, Norwich Research Park, Norwich, Norfolk NR4 7TJ, UK; 3 Cefas, Lowestoft, Suffolk NR33 0HT, UK; 4 School of Biological Sciences, Bangor University, Bangor, Gwynedd LL57 2UW, UK

**Keywords:** jellyfish, diet, gut content analysis, predation, 16S mtDNA

## Abstract

Localized outbreaks of jellyfish, known as blooms, cause a variety of adverse ecological and economic effects. However, fundamental aspects of their ecology remain unknown. Notably, there is scant information on the role jellyfish occupy in food webs: in many ecosystems, few or no predators are known. To identify jellyfish consumers in the Irish Sea, we conducted a molecular gut content assessment of 50 potential predators using cnidarian-specific mtDNA primers and sequencing. We show that jellyfish predation may be more common than previously acknowledged: uncovering many previously unknown jellyfish predators. A substantial proportion of herring and whiting were found to have consumed jellyfish. Rare ingestion was also detected in a variety of other species. Given the phenology of jellyfish in the region, we suggest that the predation was probably targeting juvenile stages of the jellyfish life cycle.

## 1. Introduction

Cnidarian jellyfish (hereafter referred to as ‘jellyfish’) are a common feature of many marine ecosystems. Localized outbreaks, known as blooms, can cause negative economic and ecological effects such as fish death, interference with marine infrastructure and tourism losses [1]. Understanding the ecology of jellyfish is essential if the blooms are to be predicted and adverse effects avoided.

One area of jellyfish ecology that is poorly characterized is their role in food webs. Jellyfish have historically been viewed as trophic dead-ends, i.e. once nutrients enter jellyfish, they are lost to organisms occupying higher trophic levels [2,3]. This viewpoint may originate from difficulties observing marine interactions *in situ* and the inability of morphological gut contents analysis (GCA) to identify rapidly digested, soft-bodied organisms such as jellyfish [4]. New analytical techniques have revealed that some animals do feed on jellyfish [5–8]. However, many of these jellyfish predators are scarce and not thought to play a significant role in controlling jellyfish populations [9]. Furthermore, most of these studies have focused on single species, and therefore the extent of jellyfish predation in food webs remains unknown.

The Irish Sea makes for an excellent case study: it has experienced adverse effects from an increasing jellyfish population [10] yet, aside from small populations of leatherback turtles and sunfish [11], no predators are known. Systematically identifying predators of jellyfish is a prerequisite step before the broader role jellyfish play in ecosystems can begin to be adequately addressed. As such, the aim of this study was to identify Irish Sea jellyfish predators, using a newly developed cnidarian-specific polymerase chain reaction (PCR) assay. This approach identifies gut contents by matching amplified DNA fragments against a DNA database, circumventing issues associated with morphological GCA. Therefore, even highly digested jellyfish can still be detected.

## 2. Methods

### 2.1. Sample collection and extraction of DNA

Sample collection, processing and molecular work is detailed in full in [12]. In summary: gut samples were collected aboard the RV *Cefas Endeavour* in the eastern Irish Sea between 25 February 2008–2 March 2008, and 19 February 2009–28 February 2009. Trawling gears were deployed to capture predators from throughout the water column to maximize active predator–prey interactions. For each haul, vinyl gloves were sealed, then inserted inside an additional vinyl glove which itself was then sealed. The innermost gloves were processed as stomachs, with the outer gloves simulating a fish during dissection: this negative control was used to detect any potential contamination introduced during initial processing. Seven hundred and fifty-one and 1762 samples were collected in 2008 and 2009, respectively. The length of each sampled organism was recorded before the removal of the stomach on-board. Gloves were changed between the dissection of different species, and instruments were decontaminated between hauls with Microsol detergent to minimize the possibility of contamination. Removed stomachs were stored at −20**°**C.

DNA was extracted from the stomach contents in a molecular laboratory. Whole sprat (*Sprattus sprattus*) and shrimp (*Crangon* sp.) stomachs had DNA extracted and purified using a salt protocol, because their stomachs were small enough to avoid issues with PCR-inhibitory substances. Samples were homogenized in 300 µl of extraction buffer (30 mM Tris–HCl pH 8.0, 10 mM EDTA pH 8.0, 1% SDS), before 5 µl Proteinase K (Qiagen) was added. The samples were then incubated at 55**°**C overnight. Purification of DNA took place using a salting-out protocol [13].

All other species’ stomachs had DNA extracted using a CTAB (hexadecyltrimethylammonium bromide) method [12]. Contents were scraped out of the dissected stomachs and homogenized in autoclaved 1.5 ml Eppendorf tubes with 350 µl of 2% CTAB buffer (100 mM Tris–HCl pH 8.0, 1.4 M NaCl, 20 mM EDTA pH 8.0, 2% CTAB, 0.2% 2-mercaptoethanol), then mixed with 5 µl Proteinase K (Qiagen) and incubated at 55**°**C overnight for the sample to digest. DNA purification was performed using two choloroform-isoamyl washes followed by a sodium acetate precipitation (3M, pH 4.8). Both CTAB and salt-extracted samples were dissolved in 100 µl water and stored at −20**°**C.

### 2.2. Jellyfish group-specific primer design

Available 16S sequences of jellyfish present in UK coastal waters (electronic supplementary material, table S1) were obtained from GenBank [14] (electronic supplementary material, table S2) and aligned using MUSCLE [15] with default settings. Positions in the 16 s alignment where nucleotides were conserved among jellyfish, but different in non-gelatinous species (electronic supplementary material, table S3) were identified. Jellyfish-specific primers: SCY_16S_F4 (TTAAATGGCCGCGGTAACT) and SCY_16S_R4 (GCTCAATAGGGTCTTTTCGTCT) were designed using Primer3 [16] to amplify a 135 bp fragment that included the unique jellyfish sequences. The primers were tested *in silico*, on non-gelatinous species (electronic supplementary material, table S3), using Amplify4 [17] prior to PCR validation across a panel of jellyfish and non-gelatinous marine species (electronic supplementary material, table S4) to ensure specificity to jellyfish.

### 2.3. Polymerase chain reaction amplification and sequencing

PCRs were conducted in 10 µl reactions containing 1 µl DNA, 1 µl 10× ReddyMix PCR Buffer IV (ABgene), 1 µl dNTPs (2 mM), 0.05 µl Thermoprime plus Taq DNA polymerase (5 U µl^−1^) (Thermo Scientific), 1 µl of Scy_16s_F1 and Scy_162_F2 (10 µM), 1.2 µl BSA (20 mg ml^−1^) (New England Biolabs), 0.6 µl MgCl_2_ (25 mM) (Thermo Scientific) and 3.15 µl H_2_O. Cycling conditions were: 95°C for 4 min, followed by 35 cycles of 95°C for 30 s, 65°C for 30 s and 72°C for 30 s, with a 10 min incubation at 72°C. Negative and positive controls were included on each plate. The presence of jellyfish DNA was determined based on the presence of a band at 177 bp on 1.5% ethidium-bromide-stained agarose gels.

A subsample of positive amplifications were purified with Exo1 (Thermo Scientific) and FastAP (Thermo Scientific) prior to Sanger sequencing (Eurofins UK). Sequences were trimmed of primers and low read-quality bases, and chromatograms visually inspected for quality. Sequences were identified using nucleotide megablast [18] against the GenBank nucleotide database, and reported as % BLAST identity values.

## 3. Results

### 3.1. 2008 Survey

Jellyfish mtDNA was detected in 18 out of 751 samples from nine of the 34 surveyed taxa (table 1). All positive samples were identified as moon jellyfish (*Aurelia aurita*) (electronic supplementary material, table S5). Five sequences from dab (*Limanda limanda*), whiting (*Merlangius merlangus*), herring (*Clupea harengus*) and squid (*Loligo* sp.) had a 100% identity match with moon jellyfish across the 135 bp amplicon. The remaining sequences also matched with moon jellyfish, but with BLAST identity values varying from 85 to 96%.

**Table 1.** Taxa tested for jellyfish feeding events.

	2008			2009		
taxa	stomachs screened	stomachs with jellyfish consumption detected	frequency of occurrence (%)	stomachs screened	stomachs with jellyfish consumption detected	frequency of occurrence (%)
*Agonus cataphractus*	1	0	0	13	0	0
*Ammodytes marinus*	0	0	n.a.	4	0	0
*Arnoglossus* sp.	0	0	n.a.	28	0	0
*Aspitrigla cuculus*	4	0	0	9	0	0
*Blennius ocellaris*	0	0	n.a.	4	0	0
*Buglossidium luteum*	0	0	n.a.	14	0	0
Callionymidae sp.	12	4	33.3	30	0	0
*Cancer pagurus*	0	0	n.a.	3	0	0
*Ciliata mustela*	2	0	n.a.	0	0	n.a.
*Clupea harengus*	143	2	1.4	369	102	27.6
*Corystes cassivelaunus*	0	0	n.a.	21	0	0
*Crangon crangon*	9	0	0	0	60	0
*Cyclopterus lumpus*	0	0	n.a.	1	0	0
*Echiichthys vipera*	13	0	0	22	0	0
*Engraulis encrasicolus*	3	0	0	0	0	n.a.
*Eutrigla gurnardus*	31	1	3.2	31	0	0
*Gadus morhua*	3	0	0	2	0	0
*Hippoglossoides platessoides*	2	0	0	0	0	n.a.
*Limanda limanda*	70	1	1.4	171	1	0.6
*Liocarcinus depurator*	0	0	n.a.	25	0	0
*Liparis liparis*	0	0	n.a.	2	0	0
*Loligo* sp.	36	1	2.8	1	0	0
Majidae sp.	0	0	n.a.	20	0	0
*Melanogrammus aeglefinus*	13	0	0	0	0	n.a.
*Merlangius merlangus*	76	2	2.6	294	34	11.6
*Microchirus variegatus*	0	0	n.a.	18	0	0
*Microstomus kitt*	0	0	n.a.	7	0	0
*Necora puber*	1	0	0	0	0	n.a.
*Nephrops norvegicus*	12	0	0	0	0	n.a.
Octopodidae sp.	0	0	n.a.	1	0	0
*Pagurus cuanensis*	0	0	n.a.	45	0	0
*Palaemon serratus*	0	0	n.a.	2	0	0
*Pandalus* sp.	1	0	0	0	0	n.a.
*Platichthys flesus*	22	0	0	39	1	2.6
*Pleuronectes platessa*	8	0	0	0	0	n.a.
*Polybius holsatus*	15	0	0	0	0	n.a.
*Pomatoschistus* sp.	1	0	0	10	0	0
*Raja clavata*	7	0	0	0	0	n.a.
*Raja montagui*	0	0	n.a.	12	0	0
*Scomber scombrus*	2	0	0	17	0	0
*Scyliorhinus canicula*	16	2	12.5	11	0	0
*Sepia officinalis*	3	0	0	0	0	n.a.
*Sepiola atlantica*	1	0	0	21	0	0
*Solea solea*	0	0	n.a.	25	1	4
*Sprattus sprattus*	192	4	2.1	412	1	0.2
*Trachurus trachurus*	4	0	0	0	0	n.a.
*Trigla lucerna*	7	0	0	8	0	0
*Trisopterus esmarkii*	10	0	0	0	0	n.a.
*Trisopterus luscus*	1	0	0	0	0	n.a.
*Trisopterus minutus*	30	1	3.3	10	0	0

### 3.2. 2009 Survey

Cnidarian mtDNA was detected in 141 samples out of 1762 samples from seven of the 38 surveyed taxa (table 1). Predation was much more frequent in herring and whiting than in 2008: jellyfish were detected in 27.6% and 11.6% of herring and whiting stomachs, respectively, compared to just 1.4% and 2.6% observed in stomachs from 2008. Samples from Dover sole (*Solea solea*), sprat and a subsample of herring (*n* = 15) and whiting (*n* = 21) were successfully sequenced. A sequence could not be obtained from the flounder (*Platichthys flesus*) amplicon; consequently, flounder was not included in further analysis. Twelve sequences from herring stomach samples were identified as moon jellyfish with three unidentified sequences. Whiting had mainly consumed mauve-stinger jellyfish (*Pelagia noctiluca*) (*n* = 16), although three mtDNA sequences derived from whiting stomachs were identified as oaten-pipe hydroids (*Tubularia indivisa*) (100% match), one sample as soft coral (*Alcyonium* sp.) (99% match), and one unidentified sequence. In contrast with 2008, most samples had 98%+ identity match (electronic supplementary material, table S5).

## 4. Discussion

### 4.1. Jellyfish consumption among common species

**Figure 1.**
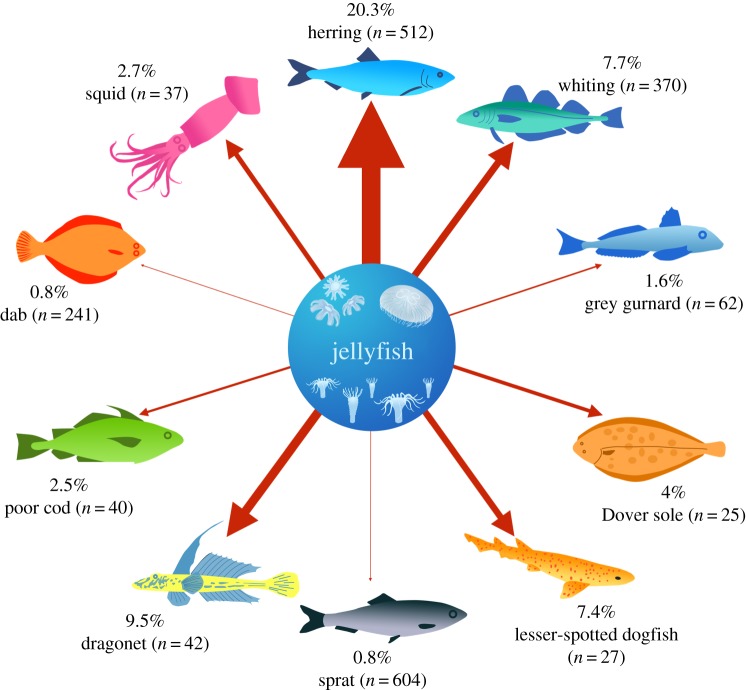
Species that feed on jellyfish in the Irish Sea validated using sequencing. Thickness of arrow is representative of the percentage of stomachs jellyfish were detected in (also displayed as a percentage) across the years 2008–2009. Reported sample sizes (*n*) refer to the number of stomachs sampled from each species. Species that jellyfish were not detected in are detailed in table 1.

Dragonet (Callionymidae sp.), grey gurnard (*Eutrigla gurnardus*), poor cod (*Trisopterus minutus*), lesser-spotted dogfish (*Scyliorhinus canicula*), squid, herring, whiting, Dover sole and sprat were identified as taxa that consume jellyfish (figure 1). High year-to-year variability was seen, particularly for whiting and herring. It is unclear what drove this variation, particularly without data for jellyfish abundance or alternative food sources for the species. However, this does highlight the importance of repeated sampling: had we formed our data from a single year, some predation events would have been missed, while other estimates would have been more inaccurate.

Jellyfish predation in the Irish Sea is not novel: sunfish and leatherback turtles are known predators [11]. However, the relative biomass of these species is tiny relative to the taxa described here (electronic supplementary material, table S6). The discovery that common species prey on jellyfish is unexpected because jellyfish predators were thought to be scarce [9]. This could be important because even apparently low levels of jellyfish consumption among common species could potentially exert comparable, or greater, levels of influence on jellyfish populations than rare predators.

### 4.2. Which species and life-history stages are being targeted?

Moon and mauve-stinger jellyfish were both found to be consumed in this study. Non-exact matches (85–96% BLAST identity) could be a result of intraspecific variation, un-sequenced cryptic species or other jellyfish absent from GenBank. The amplification of oaten-pipes hydroid and soft coral demonstrates that the primers also amplify non-scyphozoan cnidarians (some of which are jellyfish [19]), highlighting the importance of a post-PCR sequencing step to identify and remove any false positives.

Moon jellyfish possess a meta-genetic life cycle, characterized by functionally different life stages [20]. In the autumn, adult jellyfish (medusae) reproduce sexually: fertilized planktonic planulae are released, and spend several days in the water column [21] before settling on hard substrata to form sessile polyps. Medusae then begin to die off, while polyps overwinter [20]. Polyps are sessile until strobilation (the asexual production of free-swimming ephyrae) is stimulated by the onset of cooler temperatures [22] in February, March and April [23]. Ephyrae continue to develop in size, becoming medusae in around four weeks [24]. Mauve-stinger jellyfish have a similar life history, although notably lack a polyp life stage [25]. Predation on different life stages could have varying effects on jellyfish populations, and the nutrients available to predators. At the time of sample collection (February and March), the majority of moon and mauve-stinger jellyfish would not have yet matured into medusae [20]: it therefore seems probable the detected predation was on juvenile ephyrae or perhaps moon jellyfish polyps.

### 4.3. A molecular approach: advantages and limitations

A variety of approaches have been used to detect jellyfish predation. Multiple studies have identified jellyfish predation using morphological GCA approaches [5]. Shortcomings of this technique, such as systematically underestimating soft-bodied prey and taxonomic uncertainty, are well documented [4]. Recently, video loggers recorded benthic scavenging of jellyfish carcasses [26]. However, the static nature of video cameras means capturing mid-water interactions, where jellyfish spend most of their life cycle, is logistically extremely challenging. Stable isotope analysis (SIA) [6] is free of the limitations of both morphological GCA and visual observation, and also has the advantage that it provides an estimate of biomass consumed. However, SIA cannot elucidate interspecific relationships to a fine taxonomic resolution due to overlap in isotopic values between different species [27], nor is it effective at detecting rare prey species in the diet.

Molecular GCA also overcomes the limitations of morphological GCA and observational approaches, and has been used with high throughput sequencing (HTS) to identify jellyfish predation in herring in coastal waters of New Brunswick [8]. Additionally, unlike SIA, it inexpensively provides species-specific identifications. Consequently, large sample sizes can be investigated which, in this instance, proved essential to detect jellyfish consumption.

Molecular GCA does have limitations: unlike SIA, molecular GCA cannot provide biomass consumption estimates. In addition, although not widely discussed, the possibility of contamination from eDNA (trace DNA found in the environment [28]) could exist, though it is typically found at very low concentrations. In this instance, it is extremely unlikely to be problematic: in an eDNA study of Japanese sea nettle jellyfish (*Chrysaora pacifica*), the highest concentration of eDNA, detected on the sea floor, had a concentration of 2.49 × 10^−10^ ng µl^−1^ [29]. The primers used here detected moon jellyfish DNA diluted to a concentration of 0.03 ng µl^−1^, but no further. A related issue using molecular approaches is that secondary predation (when a consumed prey species has consumed jellyfish) cannot be distinguished from direct consumption of jellyfish. This is particularly problematic if using HTS: the high sensitivity makes the probability of detecting small amounts of DNA from secondary predation more likely than using the gel-imaging approach used here [30]. Secondary predation can be diagnosed by identifying predatory species in the gut alongside the jellyfish, then independently testing those species for jellyfish predation. However, by using cnidarian-specific primers, the co-occurrence of other non-cnidarian species in the guts cannot be examined; so the possibility of secondary predation should not be disregarded. Balancing the requirements of precision, cost and time needs to be carefully considered when choosing between dietary assessment methodologies: the technique employed here is fast, easy to conduct and inexpensive, but lacks the precision and sensitivity of HTS, or biomass estimates of SIA. Therefore, it is best used as a low-cost diagnostic tool for initial screening of samples to aid in the design of HTS studies, or as a complementary analysis to provide finer taxonomic resolution to SIA.

## 5. Conclusion

The evidence presented here refutes the notion that jellyfish predation is rare: sequencing suggests that herring and whiting frequently feed on jellyfish. Dragonet, sprat, Dover sole, dab, squid, lesser-spotted dogfish and poor cod were also seen to infrequently ingest jellyfish. When considering phenology of jellyfish in this region [20], it seems probable this predation is targeting juvenile jellyfish, although ingestion of moon jellyfish polyps also remains a possibility. Quantifying such feeding relationships, and testing for adult jellyfish predation later in the year are therefore important future foci towards understanding the trophic role jellyfish play in ecosystems and predicting jellyfish blooms.

## Supplementary Material

Sample details

## Supplementary Material

Stomach Sequences

## Supplementary Material

Supplementary Tables

## Data accessibility

Raw data are uploaded in the electronic supplementary material.
