# Supplementary material for: Jellyfish on the menu: mtDNA assay reveals scyphozoan predation in the Irish Sea
Source: R Soc Open Sci. 2017 Nov 29;4(11):171421. doi: 10.1098/rsos.171421 (PMC5717700; doi:10.1098/rsos.171421)
Supplement: Supplementary Tables [file rsos171421supp3.docx]

**Supplementary Table 1**. Jellyfish species present in the British Isles[1–5].

| **Genus** | **Species** |
| --- | --- |
| *Aegina* | *citrea* |
| *Aeginura* | *grimaldii* |
| *Aequorea* | *forskalea* |
| *Aequorea* | *vitrina* |
| *Aequorea* | *pensilis* |
| *Aglantha* | *digitale* |
| *Amphinema* | *dinema* |
| *Amphinema* | *rugosum* |
| *Annatiara* | *affinis* |
| *Atolla* | *wyvillei* |
| *Atolla* | *vanhoeffeni* |
| *Atolla* | *parva* |
| *Aurelia* | *aurita* |
| *Botrynema* | *brucei* |
| *Bougainvillia* | *britannica* |
| *Bougainvillia* | *macloviana* |
| *Bougainvillia* | *principis* |
| *Bougainvillia* | *ramosa* |
| *Bougainvillia* | *pyramidata* |
| *Bougainvillia* | *superciliaris* |
| *Bythotiara* | *murrayi* |
| *Chromatonema* | *rebrum* |
| *Chrysaora* | *hysocella* |
| *Cladonema* | *radiatum* |
| *Clytia* | *hemisphaerica* |
| *Clytia* | *islandica* |
| *Codonium* | *proliferum* |
| *Colobonema* | *sericeum* |
| *Corymorpha* | *nutans* |
| *Coryne* | *eximia* |
| *Cosmetira* | *pilosella* |
| *Craspedacusta* | *sowerbii* |
| *Crossota* | *rufobrunnea* |
| *Cyanea* | *capillata* |
| *Cyanea* | *lamarckii* |
| *Dipleurosoma* | *typicum* |
| *Ectopleura* | *dumortierii* |
| *Eirene* | *viridula* |
| *Eleutheria* | *dichotoma* |
| *Eucheilota* | *maculata* |
| *Eucodonium* | *brownei* |
| *Euphysa* | *aurata* |
| *Eutima* | *gegenbauri* |
| *Eutima* | *gracilis* |
| *Eutonina* | *indicans* |
| *Gonionemus* | *vertens* |
| *Halicreas* | *minimum* |
| *Haliscera* | *bigelowi* |
| *Halopsis* | *ocellata* |
| *Helgicirrha* | *schulzei* |
| *Hybocodon* | *prolifer* |
| *Laodicea* | *undulata* |
| *Leuckartiara* | *nobilis* |
| *Leuckartiara* | *octona* |
| *Liriope* | *tetraphylla* |
| *Lizzia* | *blondina* |
| *Lovenella* | *clausa* |
| *Margelopsis* | *haeckelii* |
| *Melicertum* | *octocostatum* |
| *Mitrocomella* | *brownei* |
| *Mitrocomella* | *polydiademata* |
| *Modeeria* | *rotunda* |
| *Muggiaea* | *atlantica* |
| *Nanomia* | *cara* |
| *Nausithoe* | *atlantica* |
| *Nausithoe* | *globifera* |
| *Neoturris* | *breviconis* |
| *Neoturris* | *pileata* |
| *Obelia* | *geniculata* |
| *Obelia* | *dichotoma* |
| *Obelia* | *longissima* |
| *Obelia* | *lucifera* |
| *Obelia* | *nigra* |
| *Octophialucium* | *funerarium* |
| *Orthropyxis* | *integra* |
| *Pandea* | *conica* |
| *Pandea* | *rubra* |
| *Pantachogon* | *haeckeli* |
| *Paraphyllina* | *ransoni* |
| *Pelagia* | *noctiluca* |
| *Periphylla* | *periphylla* |
| *Phialella* | *quadrata* |
| *Phialopsis* | *diegensis* |
| *Physalia* | *physalis* |
| *Podocoryna* | *borealis* |
| *Podocoryna* | *carnea* |
| *Podocoryna* | *areolata* |
| *Podocoryna* | *minima* |
| *Proboscidactyla* | *stellata* |
| *Rathkea* | *octopunctata* |
| *Rhizostoma* | *pulmo* |
| *Rhopalonema* | *velatum* |
| *Rhopalonema* | *funerarium* |
| *Sarsia* | *tubulosa* |
| *Slabberia* | *halterata* |
| *Solmaris* | *corona* |
| *Solmissus* | *incisa* |
| *Stauridiosarsia* | *gemmifera* |
| *Stauridiosarsia* | *producta* |
| *Stauridiosarsia* | *ophiogaster* |
| *Staurostoma* | *mertensii* |
| *Tetraplatia* | *volitans* |
| *Thamnostominae* | *sp.* |
| *Tiaropsis* | *multicirrata* |
| *Tima* | *bairdii* |
| *Trichydra* | *pudica* |
| *Turritopsis* | *nutricula* |
| *Velella* | *velella* |
| *Zanclea* | *costata* |

**Supplementary Table 2**. Jellyfish 16S sequences, from GenBank, used for designing group-specific jellyfish primers

| **Species** | **Accession Number** |
| --- | --- |
| *Aurelia aurita* | U19373.1 |
| *Atolla vanhoeffeni* | JX393250.1 |
| *Atolla wyvillei* | JX393251.1 |
| *Cyanea capillata* | KM114287.1 |
| *Pelagia noctiluca* | JX393260.1 |

**Supplementary Table 3**. Non-gelatinous species 16S sequences, from GenBank, used for designing group-specific jellyfish primers

| **Species** | **Accession Number** |
| --- | --- |
| *Actinia equina* | KP090930.1 |
| *Aequipecten opercularis* | AJ245397.1 |
| *Alcyonium digitatum* | AF530482.1 |
| *Antedon bifida* | KC626604.1 |
| *Bispira porifera* | HM800968.1 |
| *Buccinum pemphigus* | FJ875946.1 |
| *Buglossidium luteum* | KJ128718.1 |
| *Crangon crangon* | EU868649.1 |
| *Inachus dorsettensis* | KC866331.1 |
| *Labrus bergylta* | KJ128797.1 |
| *Limanda limanda* | AY368897.1 |
| *Liocarcinus holsatus* | GQ268540.1 |
| *Lophius piscatorius* | KJ128815.1 |
| *Luidia sarsi* | AY652495.1 |
| *Microchirus variegatus* | FN688074.1 |
| *Mullus surmuletus* | KJ128836.1 |
| *Munida* sp. | AY351197.1 |
| *Nemertesia antennina* | FJ550458.1 |
| *Ophiura ophiura* | AY652508.1 |
| *Pandalus montagui* | EU868698.1 |
| *Pasiphaea telacantha* | KP725635.1 |
| *Processa guyanae* | EU868708.1 |
| *Psammechinus miliaris* | AY652516.1 |
| *Trisopterus minutus* | KJ128939.1 |
| *Trisopterus luscus* | KJ128937.1 |
| *Tritonia plebeia* | AJ223393.1 |

**Supplementary Table 4**. Jellyfish and non-gelatinous species used to test and optimise group-specific primers on

| **Taxa** | **Grouping** |
| --- | --- |
| *Aequorea sp.* | Jellyfish |
| *Aurelia aurita* | Jellyfish |
| *Chrysaora hysoscella* | Jellyfish |
| *Cyanea lamarckii* | Jellyfish |
| *Pelagia noctiluca* | Jellyfish |
| *Rhizostoma pulmo* | Jellyfish |
| Actiniaria sp. | Non-gelatinous |
| *Aequipecten opercularis* | Non-gelatinous |
| *Alcyonium digitatum* | Non-gelatinous |
| *Antedon bifida* | Non-gelatinous |
| *Arnoglossus laterna* | Non-gelatinous |
| *Ascidiella scabra* | Non-gelatinous |
| *Buccinum sp.* | Non-gelatinous |
| *Buglossidium luteum* | Non-gelatinous |
| *Cellaria sp.* | Non-gelatinous |
| *Cirolana sp.* | Non-gelatinous |
| *Crangon allmani* | Non-gelatinous |
| *Hyalonema sp.* | Non-gelatinous |
| *Hyperoplus immaculatus* | Non-gelatinous |
| *Inachus dorsettensis* | Non-gelatinous |
| *Labrus bergylta* | Non-gelatinous |
| *Limanda limanda* | Non-gelatinous |
| *Liocarcinus holsatus* | Non-gelatinous |
| *Loligo forbesii* | Non-gelatinous |
| *Lophius piscatorius* | Non-gelatinous |
| *Luidia sarsii* | Non-gelatinous |
| *Microchirus variegatus* | Non-gelatinous |
| *Mullus surmuletus* | Non-gelatinous |
| *Munida rugosa* | Non-gelatinous |
| *Nemertesia sp.* | Non-gelatinous |
| *Ophiura ophiura* | Non-gelatinous |
| *Pagurus prideaux* | Non-gelatinous |
| *Pandalus sp.* | Non-gelatinous |
| *Pasiphaea sp.* | Non-gelatinous |
| Porifera sp. | Non-gelatinous |
| *Processa sp.* | Non-gelatinous |
| *Psammechinus miliaris* | Non-gelatinous |
| *Pycnogonum sp.* | Non-gelatinous |
| *Scalpellum sp.* | Non-gelatinous |
| *Symphodus melops* | Non-gelatinous |
| *Thyone sp.* | Non-gelatinous |
| *Trisopterus luscus* | Non-gelatinous |
| *Trisopterus minutus* | Non-gelatinous |
| *Tritonia hombergii* | Non-gelatinous |
| *Zeugopterus regius* | Non-gelatinous |

**Supplementary Table 5.** Sequencing a subsample of positive results identifies, via a megablast query on GenBank, which gelatinous species was present in the samples.

| **Species** | **Jellyfish identity** | **Blast Identity (%)** | **Year** |
| --- | --- | --- | --- |
| *Callionymidae sp.* | *Aurelia aurita* | 90 | 2008 |
| *Callionymidae sp.* | *Aurelia aurita* | 92 | 2008 |
| *Callionymidae sp.* | *Aurelia aurita* | 93 | 2008 |
| *Callionymidae sp.* | *Aurelia aurita* | 85 | 2008 |
| *Clupea harengus* | *Aurelia aurita* | 87 | 2008 |
| *Clupea harengus* | *Aurelia aurita* | 100 | 2008 |
| *Eutrigla gurnardus* | *Aurelia aurita* | 88 | 2008 |
| *Limanda limanda* | *Aurelia aurita* | 100 | 2008 |
| *Limanda limanda* | *Aurelia aurita* | 85 | 2008 |
| *Limanda limanda* | *Aurelia aurita* | 89 | 2008 |
| *Loligo sp.* | *Aurelia aurita* | 100 | 2008 |
| *Merlangius merlangus* | *Aurelia aurita* | 87 | 2008 |
| *Merlangius merlangus* | *Aurelia aurita* | 96 | 2008 |
| *Merlangius merlangus* | *Aurelia aurita* | 100 | 2008 |
| *Scyliorhinus canicula* | *Aurelia aurita* | 92 | 2008 |
| *Scyliorhinus canicula* | *NA* | NA | 2008 |
| *Sprattus sprattus* | *Aurelia aurita* | 89 | 2008 |
| *Sprattus sprattus* | *NA* | NA | 2008 |
| *Sprattus sprattus* | *Aurelia aurita* | 94 | 2008 |
| *Trisopterus minutus* | *Aurelia aurita* | 100 | 2008 |
| *Clupea harengus* | *NA* | NA | 2009 |
| *Clupea harengus* | *Aurelia aurita* | 95 | 2009 |
| *Clupea harengus* | *NA* | NA | 2009 |
| *Clupea harengus* | *Aurelia aurita* | 100 | 2009 |
| *Clupea harengus* | *NA* | NA | 2009 |
| *Clupea harengus* | *Aurelia aurita* | 100 | 2009 |
| *Clupea harengus* | *Aurelia aurita* | 100 | 2009 |
| *Clupea harengus* | *Aurelia aurita* | 95 | 2009 |
| *Clupea harengus* | *Aurelia aurita* | 98 | 2009 |
| *Clupea harengus* | *Aurelia aurita* | 100 | 2009 |
| *Clupea harengus* | *Aurelia aurita* | 99 | 2009 |
| *Clupea harengus* | *Aurelia aurita* | 100 | 2009 |
| *Clupea harengus* | *Aurelia aurita* | 100 | 2009 |
| *Clupea harengus* | *Aurelia aurita* | 100 | 2009 |
| *Clupea harengus* | *Aurelia aurita* | 100 | 2009 |
| *Clupea harengus* | *Aurelia aurita* | 100 | 2009 |
| *Merlangius merlangus* | *Alcyonium sp.* | 99 | 2009 |
| *Merlangius merlangus* | *Pelagia noctiluca* | 100 | 2009 |
| *Merlangius merlangus* | *Pelagia noctiluca* | 100 | 2009 |
| *Merlangius merlangus* | *Pelagia noctiluca* | 92 | 2009 |
| *Merlangius merlangus* | *Pelagia noctiluca* | 100 | 2009 |
| *Merlangius merlangus* | *Pelagia noctiluca* | 100 | 2009 |
| *Merlangius merlangus* | *Pelagia noctiluca* | 100 | 2009 |
| *Merlangius merlangus* | *Pelagia noctiluca* | 100 | 2009 |
| *Merlangius merlangus* | *Pelagia noctiluca* | 100 | 2009 |
| *Merlangius merlangus* | *Pelagia noctiluca* | 99 | 2009 |
| *Merlangius merlangus* | *Pelagia noctiluca* | 97 | 2009 |
| *Merlangius merlangus* | *Pelagia noctiluca* | 100 | 2009 |
| *Merlangius merlangus* | *Pelagia noctiluca* | 100 | 2009 |
| *Merlangius merlangus* | *Pelagia noctiluca* | 100 | 2009 |
| *Merlangius merlangus* | *Pelagia noctiluca* | 100 | 2009 |
| *Merlangius merlangus* | *Pelagia noctiluca* | 94 | 2009 |
| *Merlangius merlangus* | *Pelagia noctiluca* | 100 | 2009 |
| *Merlangius merlangus* | *Tubularia indivisa* | 100 | 2009 |
| *Merlangius merlangus* | *Tubularia indivisa* | 100 | 2009 |
| *Merlangius merlangus* | *NA* | NA | 2009 |
| *Merlangius merlangus* | *Tubularia indivisa* | 100 | 2009 |
| *Solea solea* | *Pelagia noctiluca* | 100 | 2009 |
| *Sprattus sprattus* | *Pelagia noctiluca* | 100 | 2009 |

**Supplementary Table 6**. Biomass of jellyfish predators in the Irish Sea. This assumes the Irish Sea area is 58000 km^2^. Sources for the data used in these calculations are referenced in the relevant data cell.

|  | ***M. mola*** | ***D. coriacea*** | ***C. harengus*** | ***Callionymidae sp*** | ***M. merlangus*** | ***Eutrigla sp.*** | ***S. canicula*** | ***Trisopterus sp.*** | ***S. sprattus*** | ***S. solea*** |
| --- | --- | --- | --- | --- | --- | --- | --- | --- | --- | --- |
| **Density (individuals km^-2^)** | 0.043  [6] | 0.000775862 *[7] |  |  |  |  |  |  |  |  |
| **Body mass (kg)** | 55  **[8] | 455  [9] |  |  |  |  |  |  |  |  |
| **Predator biomass (tonnes km^-2^)** | 0.0024 | 0.0004 | 4.0415  [10] | 0.1710  [11] | 0.5070  [11] | 0.4440 [11] | 0.2880 [11] | 0.9740  [11] | 0.7378  [10] | 0.1100  [11] |
| **Total Biomass (tonnes)** | 137.17 | 20.4750 | 234409.8 | 9918.0 | 29406.0 | 25752.0 | 16704.0 | 56492.0 | 42790.5 | 6380 |

*Based on 45 Leatherback sightings, in the Irish sea in 2012

**Body mass is based on mean values obtained from Figure 3A

**References**

1. Russell, F. 1970 *The Medusae of the British Isles II*. London: Cambridge University Press.

2. Russell, F. 1953 *The Medusae of the British Isles*. London: Cambridge University Press.

3. Laakmann, S. & Holst, S. 2014 Emphasizing the diversity of North Sea hydromedusae by combined morphological and molecular methods. *Journal of Plankton Research* **36**, 64–76. (doi:10.1093/plankt/fbt078)

4. Greve, W., Reiners, F., Nast, J. & Hoffmann, S. 2004 Helgoland Roads meso- and macrozooplankton time-series 1974 to 2004: lessons from 30 years of single spot, high frequency sampling at the only off-shore island of the North Sea. *Helgoland Marine Research* **58**, 274–288. (doi:10.1007/s10152-004-0191-5)

5. Pikesley, S., Godley, B., Ranger, S., Richardson, P. & Witt, M. 2014 Cnidaria in UK coastal waters: description of spatio-temporal patterns and inter-annual variability. *Journal of the Marine Biological Association of the United Kingdom* **94**, 1401–1408. (doi:10.1017/S0025315414000137)

6. Breen, P., Cañadas, A., Cadhla, O. Ó., Mackey, M., Scheidat, M., Geelhoed, S. C. V., Rogan, E. & Jessopp, M. 2017 New insights into ocean sunfish (Mola mola) abundance and seasonal distribution in the northeast Atlantic. *Scientific Reports* **7**, 2025. (doi:10.1038/s41598-017-02103-6)

7. Penrose, R. S. & Gander, L. R. 2013 British Isles & Republic of Ireland Marine Turtle Strandings & Sightings Annual Report 2012. *Marine Environmental Monitoring* , 1–26.

8. Watanabe, Y. & Sato, K. 2008 Functional dorsoventral symmetry in relation to lift-based swimming in the ocean sunfish Mola mola. *PLoS ONE* **3**. (doi:10.1371/journal.pone.0003446)

9. Heaslip, S. G., Iverson, S. J., Bowen, W. D. & James, M. C. 2012 Jellyfish support high energy intake of leatherback sea turtles (dermochelys coriacea): Video evidence from animal-borne cameras. *PLoS ONE* **7**, 1–7. (doi:10.1371/journal.pone.0033259)

10. Pliru, A., Kooij, J. Van Der, Engelhard, G. H., Fox, C. J., Milligan, S. P. & Hunter, E. 2012 Sprat feeding behaviour, selective predation, and impact on plaice egg mortality. *ICES Journal of Marine Science* **69**, 1019–1029. (doi:10.1093/icesjms/fss081)

11. Lees, K. and M. & Mackinson, S. 2007 An Ecopath model of the Irish Sea : ecosystems properties and sensitivity. *Sci. Ser. Tech Rep., Cefas Lowestoft* **138**, 49.
